# Decreased Cardiovascular Risk after Roux-en-Y Gastric Bypass Surgery in Chinese Diabetic Patients with Obesity

**DOI:** 10.1155/2017/5612049

**Published:** 2017-07-04

**Authors:** Xue Zhao, Wenyan Duan, Chenglin Sun, Zhuo Li, Yujia Liu, Xianchao Xiao, Gang Wang, Xiaokun Gang, Guixia Wang

**Affiliations:** ^1^Department of Endocrinology and Metabolism, The First Hospital of Jilin University, Changchun, Jilin Province 130021, China; ^2^Jilin Province People's Hospital, Changchun, Jilin Province 130021, China

## Abstract

**Background:**

The influence of bariatric surgery on cardiovascular risks in Chinese diabetic patients remains unclear. Here, we aimed to explore the impact of Roux-en-Y gastric bypass surgery (RYGB) on cardiovascular risks in Chinese diabetic patients with obesity.

**Methods:**

Twenty Chinese patients with T2DM and obesity undergoing RYGB surgery were included in this study. Cardiovascular risk factors were measured before and 18 months after surgery. A 10-year cardiovascular risk was calculated by the UKPDS risk engine. Linear regression analysis was performed on CHD risk, stroke risk, and baseline metabolic parameters.

**Results:**

The complete remission rate of diabetes was 90% after RYGB surgery, with significant improvements in blood pressure, BMI, glucose, and lipid metabolism (*P* < 0.05). The 10-year cardiovascular risk of coronary heart disease reduced from 13.05% to 3.81% (*P* = 0.001) and the 10-year risk of stroke reduced from 19.66% to 14.22% (*P* = 0.002). In subgroup analysis, Chinese diabetic patients who were women, <45 years old, with BMI < 35 kg/m^2^, and DM duration > 5 years, using noninsulin therapy presented more obvious improvements in the 10-year cardiovascular risk after RYGB surgery. WHR, age, LDL-C, and HbA1c were the most important factors influencing CHD or stroke risk after RYGB surgery (*P* < 0.01).

**Conclusion:**

RYGB surgery is an effective treatment to reduce cardiovascular risk in Chinese diabetic patients with obesity.

## 1. Introduction

Diabetes mellitus has been considered as a global burden of public health with striking prevalence in the past few years [1]. Emerging evidence has shown the close relationship between diabetes and cardiovascular diseases (CVD) [2]. Also, CVD is known to make great contribution to the high mortality rate of patients with type 2 diabetes (T2DM) [3]. Thus, it becomes extremely important to perform effective prevention strategies related to CVD during management of patients with T2DM.

Cardiovascular risk factors mainly refer to hypertension, hyperlipidemia, hyperglycemia, hyperinsulinemia, and so on. These risk factors play critical roles in the incidence and development of CVD. Among available methods to reduce cardiovascular risks, losing body weight seems to be important for a full benefit [4]. Studies have shown that a 10% reduction in body weight in people with obesity can lead to significant improvements in glucose, insulin, lipid profiles, and inflammatory markers [5]. Bariatric surgery, known as one of the most effective methods to lose weight, has shown to be beneficial in decreasing cardiovascular risk factors and preventing CVD events in studies on Hispanic, Mediterranean, and Swedish patients with T2DM [6] [7]. However, there was very little available evidence in Chinese patients with T2DM and obesity on cardiovascular risk after bariatric surgery. Different from other ethnical subjects, Chinese obese patients with T2DM usually hold a smaller BMI (<35 kg/m^2^) and more obvious islet dysfunction at the early stage of T2DM. Thus, it is of great value to explore the effect of bariatric surgery on CV risk in Chinese.

Most previous studies focusing on cardiovascular risks in diabetic patients or obese patients applied Framingham risk scores to predict 10-year cardiovascular risk. Different from the traditional Framingham score [8], the UKPDS risk equations are specific for patients with T2DM, which incorporates glycemic parameters (HbA1c and duration of DM) into a model to calculate the 10-year risk of fatal and nonfatal CHD. Thus, the UKPDS risk engine tool might provide more accurate estimates and confidential power in the 10-year CHD risk for Chinese diabetic patients than traditional Framingham risk scores [9]. However, little evidence was available related to the cardiovascular risk calculated by the UKPDS risk engine tool in diabetic patients.

Based on abovementioned, we aimed to explore the impact of Roux-en-Y gastric bypass surgery on cardiovascular risks calculated by the UKPDS risk engine in Chinese diabetic patients with obesity and to observe its long-term effect at 18 months after surgery.

## 2. Methods

### 2.1. Subjects and Study Design

Twenty diabetic subjects with obesity who underwent laparoscopic RYGB surgery (LRYGB) in Jiahe Surgical Hospital were enrolled in this study. Medical history, age, body weight, body mass index (BMI), chest circumference (CC), hip circumference (HC), waist circumference (WC), waist-hip ratio (WHR), blood pressure (BP), and current medications were recorded before and after surgery. Fasting plasma glucose (FPG), fasting C-peptide (FCP), fasting insulin (FINS), 2-hour postprandial C-peptide (PCP), 2-hour postprandial glucose (PPG), 2-hour postprandial insulin (PINS) followed by oral glucose tolerance test (OGTT), HbA1c, and lipid profiles were measured preoperation and postoperation (1, 3, 6, 12, and 18 months). At the same time, we calculated the homeostasis model assessment insulin resistance (HOMA-IR) and the homeostasis model assessment *β*-cell (HOMA-*β*).

Patients with following diseases or medical histories were excluded: acute complications of T2DM, type 1 diabetes (T1DM), or latent autoimmune diabetes in adult (LADA); a mental disorder or unstable psychiatric illness; and severe alcohol or drug dependency, with a history of coronary heart disease, cerebral infarction, renal failure, heart failure, and severe hypertension, with high surgical risk (such as active ulcer), or with a medical history of gallstones and/or cholecystectomy.

According to the World Medical Association's Declaration of Helsinki, the approval from the Ethics Committee of our institution was achieved at the beginning. Also, informed consent was obtained from all participants.

### 2.2. Definitions of Diabetes, Obesity, Elevated BP, and Diabetes Remission

According to the 1999 World Health Organization criteria, patients were diagnosed with T2DM if they achieve the following values: fasting plasma glucose ≥ 7.0 mmol/L and/or 2 h plasma glucose ≥ 11.1 mmol/L.

For the diagnosis of obesity, BMI was applied for classification to complying with Working Group on Obesity in China (WGOC) standards [10]: (1) normal weight: 18.5 kg/m^2^ < BMI < 24 kg/m^2^; (2) overweight: 24 kg/m^2^ ≤ BMI < 28 kg/m^2^; and (3) obesity: BMI ≥ 28 kg/m^2^.

Elevated blood pressure was defined as systolic blood pressure (SBP) ≥ 130 mmHg or diastolic blood pressure (DBP) ≥ 85 mmHg or current treatment for hypertension based on criteria established by JCDCG [11].

The postoperative effect on T2DM was characterized as complete remission, partial remission, and no remission [12]: (1) complete remission: FPG < 7.0 mmol/L, PPG ≤ 10.0 mmol/L, and HbA1c < 6.5% for 1 year without extra medication; (2) partial remission: FPG ≥ 7.0 mmol/L (but lower than before), PPG > 10.0 mmol/L (but lower than before), or HbA1c ≥ 6.5% (but lower than before) or a decreased dosage of medication; and (3) no remission: FPG ≥ 7.0 mmol/L (higher than before), PPG > 10.0 mmol/L (higher than before), or HbA1c ≥ 6.5% (higher than before) or an increased dosage of medication.

### 2.3. Anthropometric Measurements and Laboratory Assays

Standard methods were used to measure weight, BMI, waist circumference, chest circumference, hip circumference, waist-hip ratio, and blood pressure before and after RYGB surgery. BP was measured for 3 times by the same person using a mercury sphygmomanometer (Riva-Rocci System, ERKA, Chemnitz, Germany). All biomedical examinations were applied after receipt blood samples from patients with an overnight fast (>10 hours). Glucose oxidase method was applied to measure plasma glucose concentration. We tested HbA1c and lipid profiles including serum total cholesterol (TC), serum triglyceride (TG), high-density lipoprotein cholesterol (HDL-C), and low-density lipoprotein cholesterol (LDL-C) using a ci16200 Architect automatic analyzer (Architect, Illinois, USA). C-peptide and serum insulin were measured using radioimmunoassay (RIA) (Linco Research, St Charles, MO, USA). To evaluate the insulin resistant state and *β*-cell function, we applied the following formulas: HOMA − IR = fasting insulin (mIU/L) × fasting glucose (mmol/L)/22.5 [13] and HOMA − *β* = 20 × fasting insulin (mIU/L)/[fasting glucose (mmol/L) − 3.5] [13].

### 2.4. Cardiovascular Risk Assessment

Using the UKPDS risk engine tool, we calculated the 10-year cardiovascular risk of enrolled patients at baseline and at different postoperative time points (1, 3, 6, 12, and 18 months). This tool mainly evaluates an individual's 10-year risk of coronary heart disease (CHD), fatal CHD, stroke, and fatal stroke based on categorical values, including age, gender, HbA1c, DM duration, HDL cholesterol, total cholesterol, smoking status, blood pressure, and atrial fibrillation. The risk engine is available from the Diabetes Trials Unit, Oxford University Centre for Diabetes, Endocrinology, and Metabolism (from www.dtu.ox.ac.uk/index.php?maindoc/riskengine/).

### 2.5. Statistical Analysis

Continuous variables were presented as mean ± SD or mean ± SEM. Categorical variables are presented as numbers and percentages. Continuous variables in the different groups were tested for normal distribution using the Kruskal-Wallis normality test. For the comparison of baseline and postoperative parameters, we applied a paired *t*-test for the data subject to normal distribution and the related sample Wilcoxon signed rank test for the data subject to abnormal distribution. It was considered as statistical significance if *P* < 0.05. All statistics were calculated with SPSS 21.0 (SPSS Inc., Chicago, IL, USA).

## 3. Results

Among all the 20 diabetic patients with obesity undergoing RYGB surgery, 7 were males (35%) and 13 were females (65%), with a total mean age of 42.70 ± 12.60 years old. The mean duration from the diagnosis of T2DM was 5.35 ± 2.72 years. And detailed population characteristics and medical history are shown in Table 1. The complete remission rate of T2DM was 90% (18/20) which refers to patients who can reach a target glucose level without oral hypoglycemic drug intervention after surgery for one year. The partial remission rate was 10% (2/20); these patients continued oral hypoglycemic drug treatment with a decreased dosage. Among 16 (80%) patients with elevated blood pressure at baseline, 11 of 16 (69%) patients had normalized BP after RYGB surgery and 5 of 16 patients (31%) still need antihypertension treatment, but with a decreased dosage of medication.

As shown in Table 2, clinical metabolic parameters related to adiposity, glucose metabolism, and lipid metabolism were improved significantly. BMI changed from 34.20 ± 6.22 kg/m^2^ to 25.89 ± 3.89 kg/m^2^ (18 months) with 23.41% reduction (*P* < 0.001). WHR reduced significantly from the first month to the third month after surgery (*P* = 0.048), with the final reduction of 8.08% at 18 months comparing to baseline. Besides above, SBP decreased significantly (*P* = 0.001) with 9.20% reduction while DBP performed a slighter reduction after RYGB surgery with 6.25% reduction (*P* = 0.015). Fasting glucose, insulin, C-peptide, and PPG, PINS, and PCP as well as HbA1c presented significant decreases after surgery (18 months) comparing with baseline levels (*P* < 0.01). At 18 months after surgery, there were 47.08% decrease in fasting plasma glucose (*P* < 0.001), 48.45% decrease in 2-hour postprandial glucose (*P* < 0.001), 30.53% decrease in HbA1c (*P* < 0.001), 64.56% decrease in fasting insulin (*P* < 0.001), 53.73% decrease in 2-hour postprandial insulin (*P* < 0.001), 37.30% decrease in fasting C-peptide (*P* < 0.001), and 19.59% decrease in 2-hour postprandial C-peptide (*P* = 0.002). HOMA-IR reduced significantly after RYGB surgery with 79.67% reduction (*P* < 0.001). HOMA-*β* significantly increased at the first 3 months after surgery (*P* < 0.001), then decreased subsequently to the baseline level (*P* = 0.391).

In lipid metabolism, TG and TC levels were significantly decreased, from 2.52 mmol/L (1.67, 3.88) to 1.14 mmol/L (0.81, 1.62) (*P* < 0.001) and from 6.59 mmol/L (5.17, 8.04) to 4.77 mmol/L (4.23, 5.21) (*P* < 0.001), respectively, while HDL-C increased after the surgery from 1.21 mmol/L (0.79, 1.70) to 2.25 mmol/L (1.71, 2.37) (*P* < 0.001). The decrease of TG and TC reached to 60.36% and 34.53%, respectively, and the increase of HDL-C was 70.29% after surgery (18 months). No statistical difference was found in the LDL-C level before and after surgery (18 month) (*P* = 0.642), but LDL-C showed a decrease at first 3 months and an increase thereafter.

The results on estimated 10-year cardiovascular risks calculated by the UKPDS risk engine presented significant reductions after RYGB surgery (18 months), which are shown in Table 3. The absolute and relative risks after surgery are also shown in Table 3. The risks of CHD, fatal CHD, stroke, and fatal stroke reduced 71% (*P* < 0.001), 74% (*P* < 0.001), 28% (*P* = 0.002), and 38% (*P* = 0.001), respectively.

**Figure 1:**
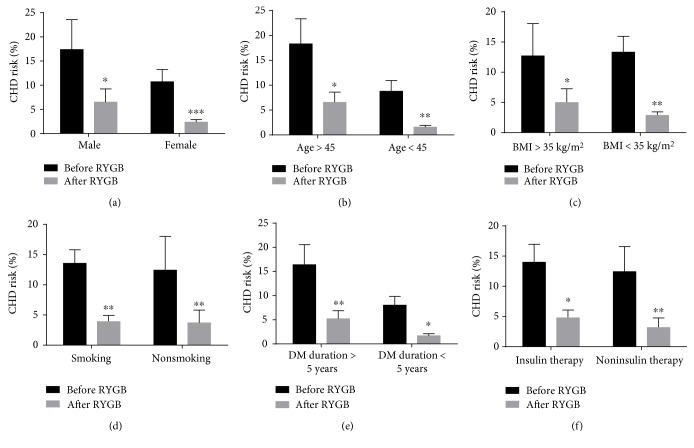
Changes of 10-year coronary heart disease (CHD) risk before and after RYGB surgery in different subgroups divided by gender, age, BMI, smoking status, DM duration, and insulin usage (baseline versus 18 months). (a) Male versus female; (b) age ≥ 45 years old versus <45 years old; (c) BMI < 35 kg/m^2^ versus BMI ≥ 35 kg/m^2^; (d) smoking versus nonsmoking; (e) DM duration ≥ 5 years versus DM duration < 5 years; and (f) insulin usage versus noninsulin usage. Data represent means ± SEM. ^∗^*P* < 0.05, ^∗∗^*P* < 0.01, and ^∗∗∗^*P* < 0.001.

Although cardiovascular risk was significantly decreased, the role of age, gender, BMI, DM duration, smoking, and insulin usage in reducing CV risk after RYGB surgery was not known. Thus, we divided the patients into different subgroups, which were classified by age (>45 years and ≤45 years), DM duration (≥5 years and <5 years), BMI (>35 kg/m^2^ and ≤35 kg/m^2^), smoking status, gender, and insulin therapy as shown in Figure 1. For CHD risk, the results showed that females could be more favorable from RYGB surgery than males (*P* = 0.001 versus *P* = 0.028). Patients who were younger than 45 years old can benefit more than people aged > 45 years old (*P* = 0.003 versus *P* = 0.011). As for BMI, patients with mild obesity (25 kg/m^2^ < BMI ≤ 35 kg/m^2^) presented more CHD risk reduced after surgery comparing those with morbid obesity (*P* = 0.003 versus *P* = 0.015). Patients with T2DM duration > 5 years can benefit more than those < 5 years through surgery (*P* = 0.003 versus *P* = 0.012). Furthermore, patients who used noninsulin therapy (oral hypoglycemic agents, exercise, and life modification) reached larger improvements than those who used insulin therapy (*P* = 0.003 versus *P* = 0.012). However, no obvious difference was found in patients with smoking habits and without smoking habits (*P* = 0.004 versus *P* = 0.008), although they both presented marked decreases in cardiovascular risk after RYGB surgery.

To find the most important factors influencing CHD and stroke risks after RYGB surgery in Chinese diabetic patients with obesity, linear regression analysis was performed (Table 4). The results which presented baseline WHR (*β*-coefficient: 0.407; *P* < 0.001), age (*β*-coefficient: 0.003; *P* < 0.001), LDL-C (*β*-coefficient: 0.017; *P* = 0.002), and HbA1c (*β*-coefficient: −0.009; *P* = 0.007) variables were related to the final CHD risk. Age (*β*-coefficient: 0.009; *P* < 0.001) and WHR (*β*-coefficient: 0.552; *P* = 0.004) were the most important factors influencing final stroke risk after surgery.

## 4. Discussion

### 4.1. Effects of RYGB Surgery on Obesity and Blood Pressure

We found twenty obese patients with T2DM who showed significant improvement in nearly all metabolic parameters related to cardiovascular risks after RYGB surgery. And eighteen of them got diabetes remission with a rate of 90%. Comparing with other studies [14, 15], our research seems to be more effective on the remission rate. It can be explained by several reasons: the lower BMI for inclusion, longer follow-up time, and different judging criteria. Most studies performed in Western countries usually apply RYGB surgery on obese T2DM patients with BMI > 35 kg/m^2^ according to standards of medical care in diabetes in 2011 from American Diabetes Association [16]. Since Chinese patients with T2DM present smaller BMI and progressing *β*-cell dysfunction at earlier time, the criteria for bariatric surgery in China are 28 kg/m^2^. Thus, it might be easier to correct metabolic disturbances for those patients with less BMI and T2DM duration. Furthermore, a longer follow-up time is necessary to detect the sustained effect of RYGB surgery on T2DM patients. However, current evidence in T2DM patients is limited and most available studies focusing on RYGB in Chinese population performed a 1-year follow-up or less. Thus, we finally analyzed a longer observation time until 18 months in order to reflect the real long-term benefits of RYGB surgery.

It is known that more than 80% of patients with T2DM are overweight or obese, and weight loss remains the hallmark in their management. RYGB surgery can help patients lose their weight quickly and sustained for a long time. In our study, patients got nearly 8.3% weight loss in the first month postsurgery (*P* < 0.001), and a long-term weight loss was also kept for 18 months reaching 25.31% weight loss finally. BMI and WHR were the known indices to evaluate adiposity and cardiovascular risks. Our results presented that RYGB surgery had obvious impacts on these variables. The possible mechanisms were unclear, mainly including decreased food intake [17], reduced absorption area after the surgery, altered gut hormones (GLP-1, PYY), gut-brain-liver axis stimulation, gut microbiota changes, and elevated bile acids [18, 19]. Although various evidences are emerging to uncover the mechanism of weight loss after RYGB surgery, it is still not clear.

Blood pressure is an important risk factor of CVD. Our results showed that RYGB surgery can significantly decrease both SBP and DBP. Moreover, SBP presented a larger reduction comparing to DBP. Similar results were also reported by Zhang et al. [20] and van Schinkel et al. [21] Thus, RYGB surgery is favorable to diabetic patients with hypertension.

### 4.2. RYGB Surgery and Glucose Metabolism as well as Lipid Metabolism

Studies have that shown bariatric surgery not only reduces the mortality related to obesity but also leads to the obviously effective glycemic control. Thus, some studies recommend this operation to be viewed as “metabolic” rather than “bariatric” surgery. In the present study, all enrolled T2DM patients had a problem in controlling their hyperglycemia at baseline. 60% patients were under treatment of oral hypoglycemic drugs, 35% patients were using insulin therapy, and the rest used the combined plan of the above two strategies. After surgery, nearly 90% patients got diabetes remission without a dosage of diabetic medications and achieved significant improvements in plasma glucose and HbA1c. Moreover, 45% patients presented hyperinsulinemia at baseline with an average level of insulin of 19.17 mU/L. Haffner et al. [22] reported that hyperinsulinemia or insulin resistance kept the central role in pathogenesis of metabolic syndrome, T2DM, and CVD. A higher HOMA-IR (≥2.6) confers higher cardiovascular risk, reported by the studies on 667 adolescents (16-17 years old) [23] and 185 Greek children [24]. Consistently, mean HOMA-IR in our patients changed from 12.19 to 2.48 with significant reduction after RYGB surgery. At the same time, the HOMA-*β* increased especially in the first 3 months after RYGB surgery indicating the marked improvement in *β*-cell function at an early stage. Similar results were also presented by Lin et al. and Camastra et al. [25, 26]. They found enhanced insulin sensitivity in specific tissues such as liver, adipose, and skeleton muscle after RYGB surgery which could sustain a long-term effect. However, the improved HOMA-*β* did not continue increasing after the first 3 months and went back to the baseline level at 18 months. This indicated that RYGB surgery could not improve the final *β*-cell function, and the initial improvement might be the result of decreased BMI.

Disturbances in lipid profiles have been shown to make great contribution in metabolic disorders in a growing body of studies and have always been linked to insulin resistance and CVD. In our study, we found significant decreases in TG and TC and increase in HDL-C after RYGB surgery with statistical difference (*P* < 0.05). These changes might be the results of improved insulin resistance and adiposity to some extent. However, there was no difference in the LDL-C level before and after surgery (*P* = 0.642). Comparing with other studies, we found that most patients in our study presented not obvious elevation in LDL-C at the baseline level which was actually at a high level of normal range. Thus, the change of LDL-C was minimal with no statistical difference after surgery.

### 4.3. RYGB and Estimated 10-Year Cardiovascular Risks

A growing body of evidence has revealed the significant improvements in cardiovascular events after bariatric surgery [27, 28]. Adams et al. [27] showed in their retrospective cohort study of 7925 matched surgical patients and obese controls that the long-term mortality was significantly reduced, especially the decrease in coronary heart disease of 56% (2.6 versus 5.9 per 10,000 person-years, *P* = 0.006). Studies have also presented the definite advantages of bariatric surgery in weight control and ameliorating cardiovascular risk factors, comparing with lifestyle modification or conventional medical therapy [7, 29]. Thus, bariatric surgery can effectively reach the goal to improve life expectancy by lowering CVD risk, which was known as the greatest threat to obese patients.

However, it usually takes too much time to monitor the end-point cardiac events, and the loss rate is another tricky problem. Thus, a better tool to predict the long-term CVD risk is required and convenient. The Framingham risk score based on Framingham Heart Study incorporates the levels of many risk factors into a single equation to produce a likelihood of CHD event in the subsequent 10 years [30]. Thereafter, in order to estimate the CVD risk specially for T2DM patients, Stevens and his colleagues presented the UKPDS risk engine tool which was suitable for T2DM patients after adding HbA1c and DM duration [8]. For a long time, the RYGB surgery was only performed in patients with morbid obesity, and the outcomes related to CVD risk in patients with mild obesity were not clear. Also, it stayed unexplored in Chinese patients with T2DM and obesity.

Using the UKPDS risk engine tool, we targeted Chinese diabetic patients undergoing RYGB surgery and found the obvious decrease in risk of CHD and stroke from 13.05% to 3.81% and 19.66% to 14.22%, respectively. The relative risk reduction reached 71% and 28%, which were consistent with another two previous studies on patients with T2DM [31, 32]. Shah et al. found that in 15 Indian diabetic patients with mild obesity, RYGB was beneficial for the glucose control and CVD risk after the 9-month follow-up [32]. In their study, the 10-year CHD risk changed from 14.9% to 4.7%, with a decrease of 69% (*P* = 0.001), and the 10-year stroke risk changed from 3.7% to 2.8%, with a decrease of 32% (*P* = 0.03). Thereafter, a larger and longer study from Cohen et al. evaluated the effects of gastric bypass surgery on 66 American patients with diabetes with encouraging results after nearly a 5-year follow-up [31]. Cohen et al. presented the significant change in the 10-year CHD risk from 35.3% to 10.3%, resolving 71% decrease (*P* = 0.001). Thus, we can believe that RYGB surgery effectively reduce the cardiovascular risk in patients with T2DM and mild obesity (BMI < 35 kg/m^2^), especially in Chinese population.

Previous studies showed that patients with diabetes had greater reduction of CHD risk than nondiabetic patients (*P* < 0.01) [33] [34]. And people who were female or >45 years old were more favorable after RYGB surgery [33, 34]. Based on above results, we further applied subgroup analysis to explore the specific effects of age, gender, BMI, DM duration, smoking status, and insulin therapy on magnitude of postoperative changes of CHD risk in Chinese population. In the present study, we found that females got a larger degree of reduce in CHD risk than males, consistent with previous studies. Different from previous studies, we found that diabetic patients who were <45 years old presented more decrease in CHD risk than older patients. The explanation for this inconsistency with a previous study might be the differences in targeted population and an estimating tool. The previous studies were mainly based on obese people > 35 kg/m^2^ with prevalence of T2DM of 28% and 38%, respectively. Furthermore, they predicted the 10-year CHD risk by the Framingham risk score, rather than the UKPDS risk engine tool. We targeted the T2DM patients with the application of the UKPDS risk engine. Thus, these differences in the study design might influence the results to some extent. Besides these, we also found diabetic patients with BMI ≤ 35 kg/m^2^ and with DM duration > 5 years using non-insulin therapy were more favorable in the 10-year CHD risk than the others. These data indicated, in Chinese population with diabetes, that BMI together with T2DM duration and diabetic therapy were also important factors to influence the final CHD risk. Also, more effective preventing strategy should be put into practice especially on diabetic patients with BMI ≤ 35 kg/m^2^ in Asian countries. And RYGB surgery seemed to present greater advantages in this population. Definitely, future studies are required to validate our findings in a larger population.

As for the factors related to CHD and stroke risks after RYGB surgery, we explored regression analysis. According to results shown in Table 4, we can conclude that WHR, age, LDL-C, and HbA1c were the most important factors. These demonstrated that adiposity, age, lipid metabolism, and glucose metabolism were related to the effect of RYGB on cardiovascular risks. On the other hand, these revealed that RYGB surgery was a more effective treatment in decreasing cardiovascular risks in diabetic Chinese by reducing weight, glucose, and lipid level at the same time comparing oral hypoglycemic medications or exercise.

## 5. Conclusions

This study presented that RYGB surgery could lead to significant decreases in cardiovascular risk factors in Chinese diabetic patients with obesity. The estimated 10-year cardiovascular risks decreased significantly at 18 months after RYGB surgery. And WHR, age, LDL-C, and HbA1c were the most important influencing factors for the reduced cardiovascular risk after RYGB surgery. In conclusion, our findings suggest that bariatric surgery is an effective treatment to reduce cardiovascular risk in Chinese diabetic patients with obesity.

## Figures and Tables

**Table 1:** Study population characteristics in T2DM patients with obesity at baseline (*n* = 20).

Variables	Before RYGB	After RYGB
Sex (M/F)	7/13	
Age (years)	42.70 ± 12.60	
Duration (year)	5.35 ± 2.72	
Smoking	9 (45%)	
OHA	12 (60%)	2 (10%)
Insulin therapy	7 (35%)	0
OHA + insulin	1 (5%)	0
Antihypertension	16 (80%)	5 (25%)

*Note*. OHA: oral hypoglycemic agent. Data represent means ± SD.

**Table 2:** Changes of cardiometabolic factors before and after RYGB (*n* = 20).

Parameters	Before RYGB	After RYGB (18 months)	*P* value
Weight (kg)	94.70 ± 21.95	70.66 ± 11.48	<0.001
BMI (kg/m^2^)	34.20 ± 6.22	25.89 ± 3.89	<0.001
WHR	1.00 ± 0.10	0.91 ± 0.09	0.002
SBP (mmHg)	136.65 ± 21.56	122.20 ± 10.18	0.001
DBP (mmHg)	85.50 ± 10.56	79.15 ± 1.16	0.015
FPG (mmol/L)	10.57 ± 3.46	5.59 ± 0.84	<0.001
PPG (mmol/L)	16.59 ± 4.31	8.55 ± 1.80	<0.001
HbA1c (%)	8.48 ± 1.49	5.89 ± 0.62	<0.001
FINS (mU/L)	19.17 (15.7, 26.8)	6.61 (5.1, 12.2)	<0.001
PINS (mU/L)	44.48 (27.2, 63.4)	24.76 (15.4, 36.6)	0.003
Fasting C-peptide (nmol/L)	1.21 ± 0.37	0.76 ± 0.23	<0.001
PCP (nmol/L)	2.52 ± 1.14	2.03 ± 0.88	0.002
HOMA-IR	8.41 (5.7, 14.3)	1.82 (1.14, 3.11)	<0.001
HOMA-beta (%)	54.83 (39.3, 100.4)	80.7 (146.8, 119.1)	0.391
TC (mmol/L)	6.59 (5.2, 8.0)	4.77 (4.2, 5.2)	<0.001
TG (mmol/L)	2.52 (1.7, 3.9)	1.14 (0.8, 1.6)	<0.001
LDL-C (mmol/L)	3.08 ± 1.15	2.94 ± 0.91	0.642
HDL-C (mmol/L)	1.21 (0.8, 1.7)	2.25 (1.7, 2.4)	<0.001

*Note*. WHR: waist-hip ratio; BMI: body mass index; SBP: systolic blood pressure; DBP: diastolic blood pressure; FPG: fasting plasma glucose; PPG: 2-hour postprandial glucose; FINS: fasting insulin; PINS: 2-hour postprandial insulin; FCP: fasting C-peptide; PCP: 2-hour postprandial C-peptide; HbA1c: hemoglobin A1c; TC: total cholesterol; TG: triglyceride; LDL-C: low-density lipoprotein cholesterol; HDL-C: high-density lipoprotein cholesterol; HOMA-IR: homeostasis model of assessment for insulin resistance index; HOMA-*β*: HOMA of beta cell function. Data represent means ± SD or M (P25, P75). *P* < 0.05: significant difference.

**Table 3:** Estimated 10-year cardiovascular risk before versus after RYGB surgery (*n* = 20).

Cardiovascular event	Presurgery mean risk (%)	Postsurgery mean risk (%)	Absolute risk reduction (%)	95% CI	Relative risk reduction (%)	*P* value
*n*	20	20				
CHD	13.05 ± 2.71	3.81 ± 1.07	9.24	(5.02, 13.46)	71	<0.001
Fatal CHD	7.09 ± 1.92	1.83 ± 0.66	5.26	(2.37, 8.14)	74	<0.001
Stroke	19.66 ± 4.46	14.22 ± 3.44	5.43	(1.72, 9.13)	28	0.002
Fatal stroke	2.86 ± 0.61	1.78 ± 0.42	1.08	(0.49, 1.66)	38	0.001

*Note*. UKPDS: United Kingdom Prospective Diabetes Study; RYGB: Roux-en-Y gastric bypass; CI: confidence interval; CHD: coronary heart disease. Data represent means ± SEM. *P* < 0.05: significant difference.

**Table 4:** Regression analysis on CHD or stroke risk (18 months) and basal clinical metabolic variables in patients with diabetes and obesity (*n* = 20).

Risks	Variables	*β*-coefficients	*P* value
CHD	WHR	0.407	<0.001
	Age	0.003	<0.001
	LDL-C	0.017	0.002

Stroke	HbA1c	−0.009	0.007
	Age	0.009	<0.001
	WHR	0.552	0.004

*Note*. CHD: coronary heart disease. *P* < 0.05: significant difference.
